# Surgical treatment of thoracolumbar spinal tuberculosis—a multicentre, retrospective, case-control study

**DOI:** 10.1186/s13018-019-1252-4

**Published:** 2019-07-23

**Authors:** Yong Tang, Wen-jie Wu, Sen Yang, Dong-Gui Wang, Qiang Zhang, Xun Liu, Tian-Yong Hou, Fei Luo, Ze-hua Zhang, Jian-zhong Xu

**Affiliations:** Department of Orthopaedics, Southwest Hospital, Third Military Medical University, No.30, Gaotanyan Street, District of Shapingba, Chongqing, 400038 China

**Keywords:** Thoracolumbar tuberculosis, Anterior-only approach, Posterior-only approach, Anterior combined with posterior approach

## Abstract

**Background:**

The purpose of this multicentre, retrospective study was to evaluate the safety and efficacy of different surgical approaches for treating thoracolumbar tuberculosis.

**Methods:**

This study reviewed 132 patients with thoracolumbar tuberculosis in six institutions between January 1999 and January 2015 surgically treated by an anterior-only approach (*n* = 22, group A), an anterior combined with posterior approach (*n* = 79, group B), and a posterior-only approach (*n* = 31, group C). All patients were treated with standard antituberculosis drugs pre- and postoperatively and were followed regularly after surgery. Clinical symptoms, nerve function, and the erythrocyte sedimentation rate were observed, and kyphosis correction and bone fusion were evaluated by X-ray or computed tomography.

**Results:**

At the last follow-up, all patients had achieved bone fusion, relief from pain, and neurological recovery. The Cobb angle was improved; however, the Cobb angle showed a degree of loss at the final follow-up after all three surgical approaches. Further comparisons revealed a difference in angle loss at the final follow-up among the three groups; groups B and C were superior to group A in maintenance of the correction. The posterior-only approach was characterized by a shorter operative time and reduced blood loss.

**Conclusions:**

Surgery by a posterior-only approach is superior to that by an anterior-only approach and anterior combined with posterior approach in terms of permanent kyphosis correction and spinal stability maintenance. Therefore, we recommend surgery by a posterior-only approach as the optimized treatment for thoracolumbar tuberculosis if the indications for this treatment are met.

## Background

The surgical strategy for treating spinal tuberculosis is to thoroughly debride tuberculosis infection lesions, carry out standardized and effective antituberculosis treatment, alleviate the symptoms of nerve compression, promote the recovery of nerve function, correct kyphosis, and rebuild spinal stability. However, there is still no consensus regarding the optimal method for surgically treating thoracolumbar tuberculosis. Previous studies have found that single-stage internal fixation via an anterior approach can achieve complete debridement, decompression, and bone graft fusion. In addition, this approach has the advantages of preventing damage to the posterior column, shortening the operative time, and promoting wound healing. Therefore, anterior single-segment fixation is a common surgical treatment for spinal tuberculosis [1, 2]. Nevertheless, some studies have reported that anterior internal fixation has no significant effect on correcting kyphosis, especially progressively aggravated kyphosis [1, 3]. Therefore, some experts recommend debridement through an anterior approach and internal fixation via a posterior approach. This treatment strategy can achieve complete debridement, ensure bone graft fusion efficacy, reduce the probability of the infection spreading, correct kyphosis, and prevent the progressive worsening of kyphosis after surgery [1, 3, 4]. However, this surgical strategy greatly increases the operative time, blood loss volume, surgical trauma, and incidence of perioperative complications [5–7]. In recent years, it has been reported that in cases of progressive single-segment spinal tuberculosis, surgery by a posterior approach can achieve complete debridement, decompression, bone fusion, and internal fixation and can effectively correct spinal kyphosis, which has the advantages of mild trauma, few perioperative complications, low cost, and short recovery time[8, 9]. To analyse the clinical efficacy of different surgical methods for the treatment of thoracolumbar tuberculosis, we retrospectively analysed 132 patients with thoracolumbar tuberculosis treated in 6 hospitals. The purpose of this study was to research the effect of different surgical approaches for curing thoracolumbar tuberculosis and provide guidelines for the selection of appropriate surgical approaches.

## Methods

Between January 1999 and January 2015, 265 patients with thoracolumbar tuberculosis were consecutively hospitalized in 6 hospitals. Patients were included in the current study if they met the following inclusion criteria: diagnosis of thoracolumbar tuberculosis (based on non-specific laboratory index and radiology), surgical treatment, no spinal tumours, good compliance, and a minimum of 18 months of postoperative follow-up. Finally, 132 patients were included in the study; there were 72 males and 60 females, with an average age of 43.55 ± 14.74 years (range, 24 to 65 years). The duration of disease was 20.05 ± 15.41 months (range, 8 to 32 months). According to the different surgical approaches, the patients were divided into the following three groups: surgery by an anterior-only approach (*n* = 22, group A), surgery by an anterior combined with posterior approach (*n* = 31, group B), and surgery by a posterior-only approach (*n* = 79, group C) (Table 1). Decisions regarding the surgical approach and instrumentation selection were made by individual surgeons at each hospital. Meanwhile, the approach selection was also related to the degree of vertebral destruction, the vertebral position, and the abscess size.

**Table 1:** General information of 132 thoracolumbar tuberculosis patients

	Group A	Group B	Group C	Statistical value
Case number	22	79	31	
M:F	12:10	40:39	16:15	*P* > 0.05
Age (years)	33.04 ± 10.21	33.45 ± 10.38	35.99 ± 10.83	*F* = 1.850, *P*_1_ = 0.834, *P*_2_ = 0.106, *P*_3_ = 0.124
Duration (months)	21.65 ± 13.34	19.62 ± 15.80	19.56 ± 16.14	*F* = 0.422, *P*_1_ = 0.980, *P*_2_ = 0.981, *P*_3_ = 0.207
Follow-up (months)	29.31 ± 7.34	29.49 ± 6.64	29.33 ± 6.91	*F* = 0.015, *P*_1_ = 0.884, *P*_2_ = 0.985, *P*_3_ = 0.878
Hospitalization (days)	27.00 ± 5.91	31.25 ± 11.69	27.72 ± 9.21	*F* = 4.012, *P*_1_ = 0.028, *P*_2_ = 0.910, *P*_3_ = 0.096
Operative time (min)	324.67 ± 44.16	422.63 ± 70.17	257.40 ± 84.01	*F* = 111.423, *P*_1_ = 0.000, *P*_2_ = 0.000, *P*_3_ = 0.000
Blood loss (ml)	895.19 ± 395.10	1187.32 ± 504.60	805.96 ± 769.58	*F* = 8.170, *P*_1_ = 0.001, *P*_2_ = 0.701, *P*_3_ = 0.000

*P*_1_: A vs B; *P*_2_: A vs C; *P*_3_: B vs C

The indications for surgery by an anterior-only approach included the following: lesion damage to the anterior and middle column of the vertebral body, penetration of the spinal canal by abscess or dead bone with symptoms of spinal cord compression, spinal kyphosis, inability to perform simple posterior segment bone grafting for bone fusion after laminectomy, and involvement of fewer than 3 vertebral bodies [10, 11]. The main indications for surgery by an anterior combined with posterior approach were the following: severe vertebral body damage or collapse; involvement of at least 3 vertebral bodies; spinal instability after debridement; invasion of the spinal canal by abscess or sequestrum, causing spinal cord compression; severe kyphosis that would be difficult to correct using an anterior or posterior approach, accompanied by a massive paravertebral, psoatic, or migrating abscess; skipped multi-segmental spinal tuberculosis; and severe spinal instability [12]. The main indications for surgery by a posterior-only approach were the following: no more than 3 adjacent vertebral segments requiring surgery; normal bone for tunnel fixation; a history of surgery by an anterior approach, which may have led to unclear anterior anatomical structures; and the ability to complete debridement through a posterior-only approach [13, 14]. The three surgical approaches were performed as standard operating procedures. There were no significant differences in the rate of the surgical approaches among the different hospitals. All patients received regular antituberculosis HREZ chemotherapy not less than 2 weeks before surgery and 18 months after surgery [15], as well as reasonably adjusted antituberculosis drugs according to the results of drug susceptibility testing [16].

Patients were required to return to the hospital for follow-up visits at 1, 3, 6, 9, and 12 months after surgery and then once per year thereafter. At each follow-up, clinical symptoms, nerve function, and the erythrocyte sedimentation rate (ESR) were recorded, and kyphosis correction and bone fusion were evaluated by X-ray or computed tomography (CT). Pain was assessed using a visual analogue scale (VAS). The Oswestry Disability Index (ODI) was used to assess improvements in disability. Neurological function was assessed by the Spinal Cord Injury Association of America (Asia) Injury Scale. The thoracolumbar angle was measured on plain lateral radiographs in each case.

All data were analysed using SPSS 19.0 statistical software. The operative time, blood loss volume, Cobb angle, VAS score, and ODI were compared by ANOVA, followed by Dunnett’s T3 test or the LSD *t* test to compare each group. Differences in gender and age were analysed the *χ*2 test and ANOVA, respectively. *P* < 0.05 was considered statistically significant.

## Results

**Fig. 1:**
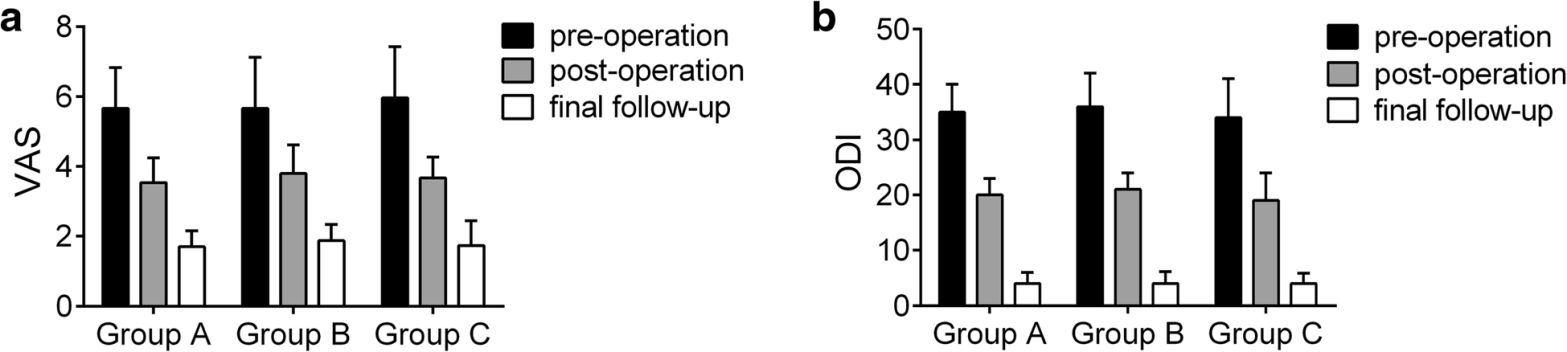
Pain and neurological function evaluation at different time in the three groups, **a**: the visual analogue scale (VAS) scores, **b**: Oswestry Disability Index (ODI) values

There were no significant differences in age, sex, or other general parameters among the three groups (*P* > 0.05). All patients were followed for no less than 18 months, with an average follow-up duration of 29.37 ± 6.90 months (range, 24 to 52 months). Group B had the largest volume of blood loss and the longest operative time (*P* < 0.05). After surgical treatment, all patients with neurological dysfunction showed significant improvements. The VAS score and ODI were significantly decreased in the 3 groups (*P* < 0.05) (Fig. 1). The ESR returned to normal levels in all patients by 3 months after surgery, and no obvious abnormalities were discovered at the last follow-up.

**Fig. 2:**
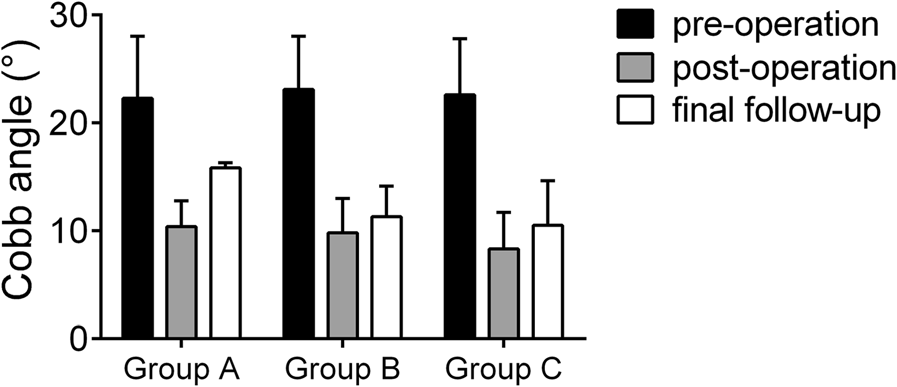
The Cobb angle at different time points in the three groups

**Fig. 3:**
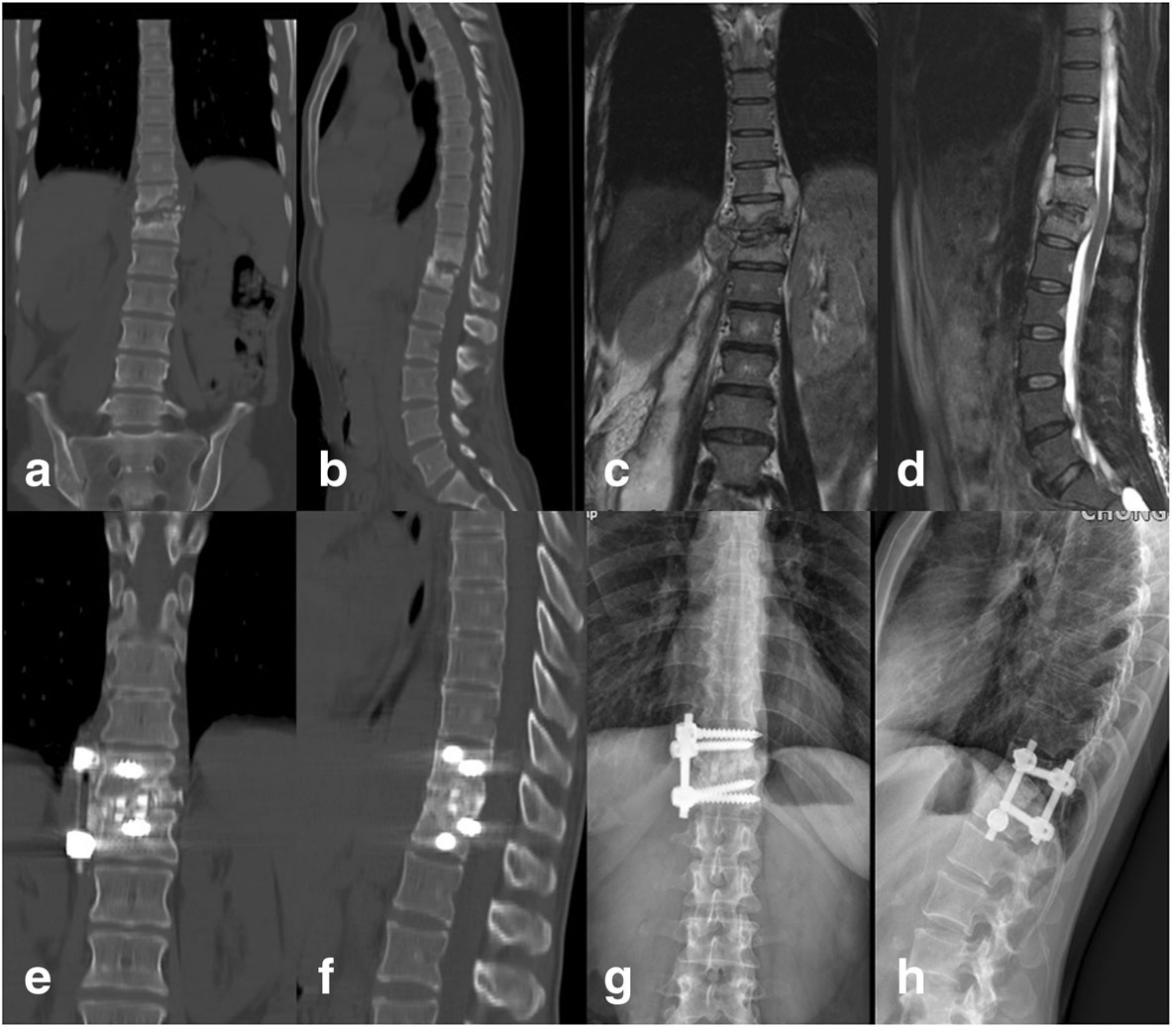
A case in which anterior debridement, bone grafting, and instrumentation were used. Illustration: the patient was a 44-year-old woman. **a**, **b** Preoperative CT. **c**, **d** Preoperative MRI. **e**, **f** CT 12 months postoperatively. **g**, **h** X-ray 12 months postoperatively

**Fig. 4:**
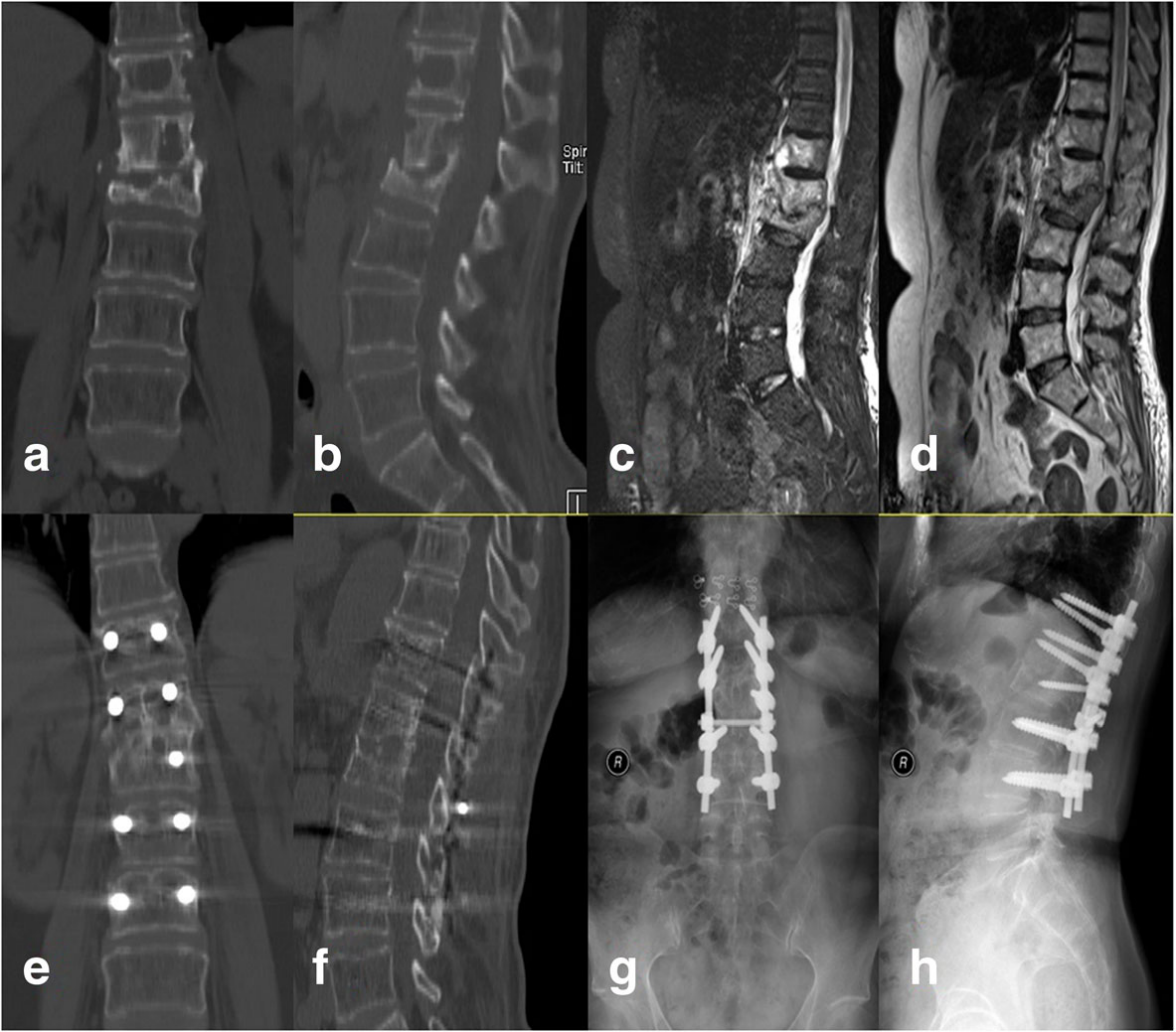
A case in which posterior debridement, bone grafting, and instrumentation were used. Illustration: the patient was a 61-year-old woman. **a**, **b** Preoperative CT. **c**, **d** Preoperative MRI. **e**, **f** CT 12 months postoperatively. **g**, **h** X-ray 12 months postoperatively

**Fig. 5:**
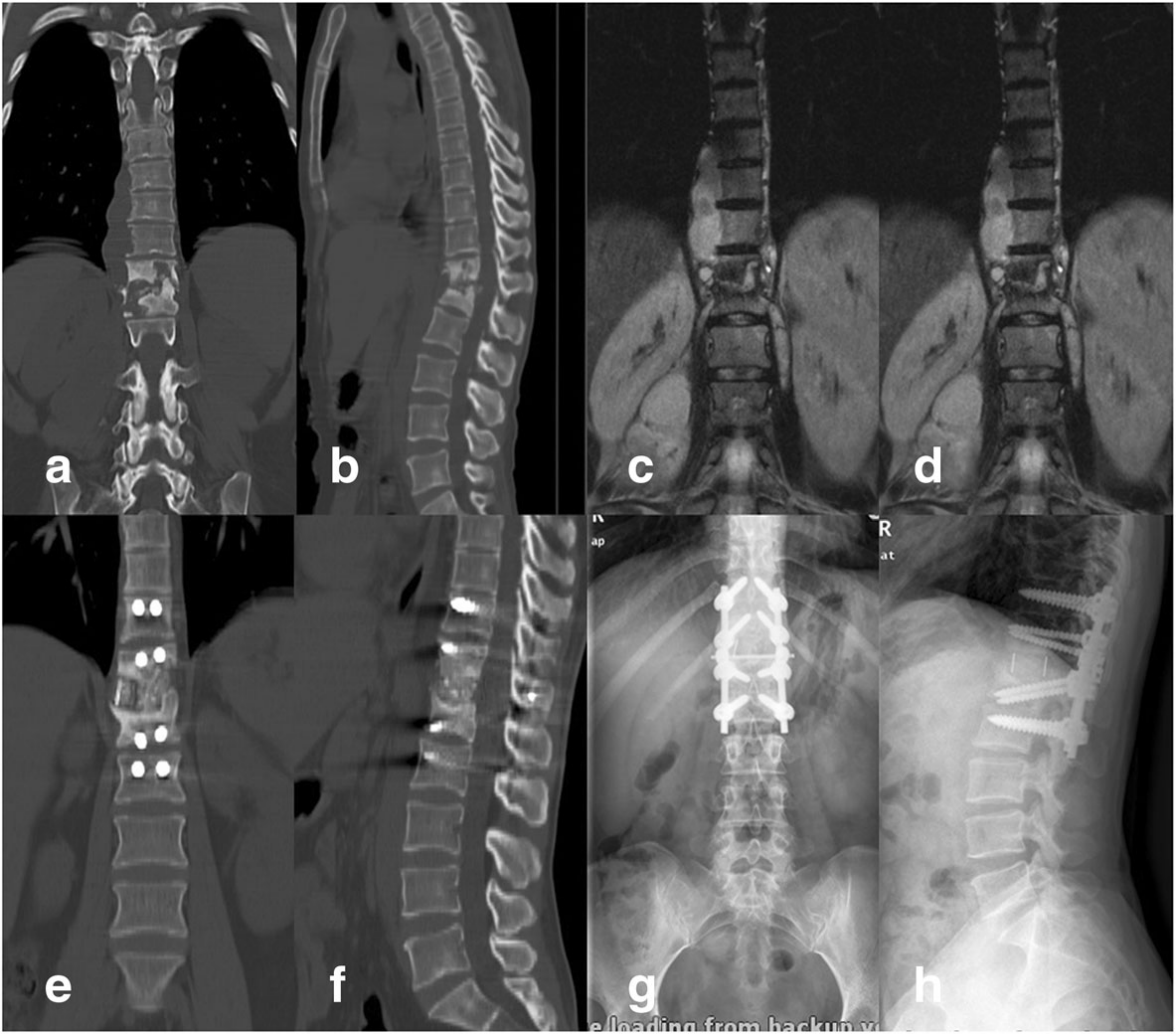
A case in which anterior debridement and bone grafting combined with posterior internal fixation were used. Illustration: the patient was a 38-year-old woman. **a**, **b** Preoperative CT; **c**, **d** Preoperative MRI. **e**, **f** CT 12 months postoperatively. **g**, **h** X-ray 12 months postoperatively

At the postoperative and final follow-ups, the average Cobb angle was 10.08 ± 4.99° and 15.54 ± 6.06°, respectively, in group A, and the Cobb angle was corrected by 5.45 ± 3.13°. The average Cobb angle was 9.11 ± 3.63° and 10.94 ± 4.03° postoperatively and at the final follow-up, respectively, in group B, and the angle was corrected by 1.82 ± 1.76°. The average Cobb angle at the postoperative and final follow-ups was 9.81 ± 5.40° and 12.01 ± 6.12°, respectively, in group C, and the angle was corrected by 2.21 ± 2.65°. Long-term follow-up revealed that the Cobb angle was significantly corrected in all three groups (*P* < 0.05). However, the angle had deteriorated by the final follow-up visit after all three surgical approaches (Fig. 2). Based on the long-term follow-up results, groups B and C showed superior results to those in group A in terms of the efficacy of deformity correction and the stability of internal fixation (Table 2). All patients achieved bone fusion within 6 to 12 months after surgery (Figs. 3, 4 and 5).

**Table 2:** Cobb angle of the patients in preoperative, postoperative, and final follow-up and loss angle

Index	Group A	Group B	Group C	Statistical value
Preoperative	20.23 ± 7.62	18.74 ± 7.31	23.21 ± 9.73	
Postoperative	10.08 ± 4.99	9.11 ± 3.63	9.81 ± 5.40	
Final follow-up	15.54 ± 6.06	10.94 ± 4.03	12.01 ± 6.12	
Loss angle (°)	5.45 ± 3.13	1.82 ± 1.76	2.21 ± 2.65	*F* = 36.615 *P*_1_ = 0.000 *P*_2_ = 0.000 *P*_3_ = 0.569
Statistical value	*F* = 33.549 *P*_1_ = 0.000 *P*_2_ = 0.002 *P*_3_ = 0.000	*F* = 67.266 *P*_1_ = 0.000 *P*_2_ = 0.000 *P*_3_ = 0.016	*F* = 104.680 *P*_1_ = 0.000 *P*_2_ = 0.000 *P*_3_ = 0.015	

*P*_a_: preoperative vs postoperative, *P*_b_: preoperative vs final follow-up, *P*_c_: postoperative vs final follow-up

The postoperative follow-up of patients in group A revealed 1 case of intercostal neuralgia, 3 cases of iliac bone donor region pain, and 2 cases of electrolyte disturbance. There were 3 cases of postoperative pectoralgia, 6 cases of iliac bone donor region pain, 5 cases of electrolyte disturbance, 1 case of intraoperative pleural injury, and 2 cases of urinary infection in group B. In group C, there were 11 cases of cerebrospinal fluid leakage, 2 cases of intercostal numbness, and 2 cases of iliac bone donor region pain. All complications were relieved after symptomatic treatment.

## Discussion

In recent years, the incidence of spinal tuberculosis has been on the rise, which has attracted the attention of researchers. There is no doubt that early diagnosis and standard antituberculosis chemotherapy are key for curing spinal tuberculosis. The thoracolumbar segments, commonly known as segments T10-L2, are the transition between the relatively fixed thoracic spine and the more active lumbar spine. In addition, this area is also the transition point from the physiological kyphosis of the thoracic spine to the physiological lordosis of the lumbar spine, with complex biomechanical characteristics. At points of stress concentration, the thoracolumbar area is vulnerable to bacterial invasion, which can cause spinal tuberculosis. Moreover, at the junction of the pleural cavity, surgery can easily damage the pleura [17]. Thoracolumbar tuberculosis mainly destroys the vertebral body, intervertebral disc, and surrounding soft tissue. It often leads to spinal kyphosis, spinal cord or nerve compression, and the loss of mechanical stability. Standard antituberculous therapy can cure most cases of spinal tuberculosis [18, 19]. However, atypical symptoms and a limited understanding of the disease lead to delays in diagnosis. Surgical treatment should be considered in the following four situations: the patient is insensitive or resistant to antituberculosis chemotherapy drugs, the patient has severe vertebral body damage, the patient has neurological dysfunction, or the patient has kyphosis or spinal instability.

Currently, there are no standard surgical approaches for treating thoracolumbar tuberculosis [20]. The surgical approaches used include the anterior, posterior, and anterior combined with posterior approaches [21]. Each approach has its advantages and disadvantages. In clinical practice, the surgical approach should be selected according to the characteristics of the lesion, the technical proficiency of the surgeon, and the imaging findings [22]. Cui et al. compared the outcomes of the anterior, anterior combined with posterior, and posterior approaches in the management of thoracolumbar tuberculosis. They found that the anterior, posterior, and combined approaches achieved good clinical effects but that posterior fixation had unique advantages in correcting kyphosis and maintaining postoperative stability [23]. However, surgery by an anterior combined with a posterior approach may increase the operative time, blood loss volume, and trauma, as well as lead to a higher incidence of perioperative complications.

Surgery by a traditional anterior-only approach has many advantages, such as wide and clear visualization of the surgical field, thorough debridement and sufficient decompression, ability to remove paravertebral and psoatic abscesses simultaneously, convenient bone grafting, and the ability to correct kyphosis and reconstruct spinal stability [24]. Surgery by an anterior-only approach also has many disadvantages, such as severe trauma, high risk of vascular injury, and loss of correction. In this study, although surgery by the anterior-only approach also corrected kyphosis, there was significant deterioration in the Cobb angle at the final follow-up, and the efficacy of this approach in terms of correction and maintenance was inferior to that of the other two approaches.

Surgery by an anterior combined with posterior approach can separate debridement from internal fixation, which is beneficial for reducing the spread of tuberculosis; meanwhile, extensive abscess debridement and spinal canal decompression can be accomplished. Furthermore, the posterior stable internal fixation system can correct spinal kyphosis, promote bone graft fusion, and maintain long-term spinal stability. However, it is not possible to avoid perioperative complications associated with surgery by an anterior approach. In addition, many studies have reported disadvantages of surgery by such a combined approach, such as an increased operative time, blood loss volume, and hospital stay [25]. In our research, the longest operative time, largest blood loss volume, and most perioperative complications were found in group B. However, the combined approach was superior to the anterior-only approach in terms of correcting kyphosis.

Surgery by a posterior-only approach is relatively simple, causes less trauma, and allows thorough debridement, fusion, and internal fixation to be achieved. There is no need to change the position of the patient during the operation, and fewer complications occur during the perioperative period. Furthermore, this approach can be used to effectively correct kyphosis and reduce internal fixation loosening and breakage. In addition, this approach reduces the operative time and blood loss volume and alleviates the economic burden. Mehta and Bhojraj have reported that the effect of surgery by a posterior-only approach for thoracolumbar tuberculosis is satisfactory [26]. Rath et al. reported that nerve function recovered well after surgery by a posterior-only approach and that the results were similar to those of surgery by an anterior-only approach [27]. Our research shows the superiority of a posterior-only approach to an anterior-only approach in terms of correcting kyphosis, possibly because of the more stable biomechanical properties of the pedicle screw system for maintaining fixation [28, 29]. However, a posterior-only approach also has some disadvantages, such as a narrow visual field, high technical requirements, dural injury, and surrounding tissue adhesion. Furthermore, a posterior approach destroys the posterior column, which has an influence on stability [30]. In this study, there was no significant difference in blood loss between the posterior and anterior approaches; we consider that this result may be related to the obvious learning curve of the posterior approach.

Our study has some shortcomings and limitations. First, this study is not a prospective cohort study and lacks a sufficiently large sample size. In addition, the follow-up time is relatively short, and long-term efficacy remains to be observed. Second, although the six hospitals noted are comprehensive teaching hospitals, the patients’ conditions and doctors’ surgical skills were different. Therefore, the advantages found in this study related to the posterior-only approach may be due to some deviations caused by the status of individual patients in the surgery and the proficiency of individual surgeons, which may lead to the decision to choose a posterior-only approach.

## Conclusions

A posterior-only approach is superior to an anterior-only approach and an anterior combined with posterior approach because it permanently corrects kyphosis and maintains spinal stability. Therefore, we recommend surgery by a posterior-only approach as the optimized treatment for thoracolumbar tuberculosis if the indications for such an approach are met.

## Data Availability

The datasets used and analyzed during the current study are available from the corresponding author on reasonable request.

## Abbreviations

ESR: Erythrocyte sedimentation rate
HREZ: Isoniazid H, rifampicin R, ethambutol E, pyrazinamide Z
ODI: Oswestry Disability Index
VAS: Visual analogue scale

## References

[1] Jain AK, Dhammi IK, Jain S, Kumar J (2010). Simultaneously anterior decompression and posterior instrumentation by extrapleural retroperitoneal approach in thoracolumbar lesions. Indian journal of orthopaedics..

[2] Rawall S, Mohan K, Nene A (2013). Posterior approach in thoracolumbar tuberculosis: a clinical and radiological review of 67 operated cases. Musculoskelet Surg.

[3] Hu J, Li D, Kang Y, Pang X, Wu T, Duan C (2014). Active thoracic and lumbar spinal tuberculosis in children with kyphotic deformity treated by one-stage posterior instrumentation combined anterior debridement: preliminary study. Eur J Orthop Surg Traumatol.

[4] Hirakawa A, Miyamoto K, Masuda T, Fukuta S, Hosoe H, Iinuma N (2010). Surgical outcome of 2-stage (posterior and anterior) surgical treatment using spinal instrumentation for tuberculous spondylitis. J Spinal Disord Tech.

[5] Zhong N, Kong J, Sun Z, Qian M, Liu T, Xiao J (2018). One-stage posterior approach in the treatment of consecutive multi-segment thoracic tuberculosis with kyphosis. Turk Neurosurg.

[6] Shi J, Tang X, Xu Y, Zhou T, Pan X, Lin H (2014). Single-stage internal fixation for thoracolumbar spinal tuberculosis using 4 different surgical approaches. J Spinal Disord Tech.

[7] Wang LJ, Zhang HQ, Tang MX, Gao QL, Zhou ZH, Yin XH (2017). Comparison of three surgical approaches for thoracic spinal tuberculosis in adult: minimum 5-year follow up. Spine..

[8] D'Souza AR, Mohapatra B, Bansal ML, Das K (2017). Role of posterior stabilization and transpedicular decompression in the treatment of thoracic and thoracolumbar TB: a retrospective evaluation. Clin Spine Surg.

[9] Hu X, Zhang H, Yin X, Chen Y, Yu H, Zhou Z (2016). One-stage posterior focus debridement, fusion, and instrumentation in the surgical treatment of lumbar spinal tuberculosis with kyphosis in children. Childs Nerv Syst.

[10] Hassan K, Elmorshidy E (2016). Anterior versus posterior approach in surgical treatment of tuberculous spondylodiscitis of thoracic and lumbar spine. Eur Spine J.

[11] Jin W, Wang Q, Wang Z, Geng G (2014). Complete debridement for treatment of thoracolumbar spinal tuberculosis: a clinical curative effect observation. Spine J.

[12] Liu J, Wan L, Long X, Huang S, Dai M, Liu Z (2015). Efficacy and safety of posterior versus combined posterior and anterior approach for the treatment of spinal tuberculosis: a meta-analysis. World Neurosurg.

[13] Wang YX, Zhang HQ, Li M, Tang MX, Guo CF, Deng A (2017). Debridement, interbody graft using titanium mesh cages, posterior instrumentation and fusion in the surgical treatment of multilevel noncontiguous spinal tuberculosis in elderly patients via a posterior-only. Injury.

[14] Guzey FK, Emel E, Bas NS, Hacisalihoglu S, Seyithanoglu MH, Karacor SE (2005). Thoracic and lumbar tuberculous spondylitis treated by posterior debridement, graft placement, and instrumentation: a retrospective analysis in 19 cases. J Neurosurg Spine.

[15] Mei G, Luo F, Zhang Z, Dai F, Zhou Q, He Q (2016). Treatment experiences and management outcomes for skipped multisegmental spinal tuberculosis. Orthopedics..

[16] Chhabra N, Gupta N, Aseri ML, Mathur SK, Dixit R (2011). Analysis of thyroid function tests in patients of multidrug resistance tuberculosis undergoing treatment. J Pharmacol Pharmacother.

[17] Kim KT, Park KJ, Lee JH (2009). Osteotomy of the spine to correct the spinal deformity. Asian Spine J.

[18] Zhang Z, Luo F, Zhou Q, Dai F, Sun D, Xu J (2016). The outcomes of chemotherapy only treatment on mild spinal tuberculosis. J Orthop Surg Res.

[19] van Loenhout-Rooyackers JH, Verbeek AL, Jutte PC (2002). Chemotherapeutic treatment for spinal tuberculosis. Int J Tuberc Lung Dis.

[20] Lee SH, Sung JK, Park YM (2006). Single-stage transpedicular decompression and posterior instrumentation in treatment of thoracic and thoracolumbar spinal tuberculosis: a retrospective case series. J Spinal Disord Tech.

[21] Sahoo MM, Mahapatra SK, Sethi GC, Dash SK (2012). Posterior-only approach surgery for fixation and decompression of thoracolumbar spinal tuberculosis: a retrospective study. J Spinal Disord Tech.

[22] Wang Z, Wu Q, Geng G (2013). Anterior debridement and bone grafting with posterior single-segment internal fixation for the treatment of mono-segmental spinal tuberculosis. Injury..

[23] Cui X, Ma YZ, Chen X, Cai XJ, Li HW, Bai YB (2013). Outcomes of different surgical procedures in the treatment of spinal tuberculosis in adults. Med Princ Pract.

[24] Jin D, Qu D, Chen J, Zhang H (2004). One-stage anterior interbody autografting and instrumentation in primary surgical management of thoracolumbar spinal tuberculosis. Eur Spine J.

[25] Memtsoudis SG, Vougioukas VI, Ma Y, Gaber-Baylis LK, Girardi FP (2011). Perioperative morbidity and mortality after anterior, posterior, and anterior/posterior spine fusion surgery. Spine..

[26] Mehta JS, Bhojraj SY (2001). Tuberculosis of the thoracic spine. A classification based on the selection of surgical strategies. J Bone Joint Surg Br.

[27] Rath SA, Neff U, Schneider O, Richter HP (1996). Neurosurgical management of thoracic and lumbar vertebral osteomyelitis and discitis in adults: a review of 43 consecutive surgically treated patients. Neurosurgery..

[28] Deniz FE, Brasiliense LB, Lazaro BC, Reyes PM, Sawa AG, Sonntag VK (2010). Biomechanical evaluation of posterior thoracic transpedicular discectomy. J Neurosurg Spine.

[29] Pu X, Zhou Q, He Q, Dai F, Xu J, Zhang Z (2012). A posterior versus anterior surgical approach in combination with debridement, interbody autografting and instrumentation for thoracic and lumbar tuberculosis. Int Orthop.

[30] Zhang H, Sheng B, Tang M, Guo C, Liu S, Huang S (2013). One-stage surgical treatment for upper thoracic spinal tuberculosis by internal fixation, debridement, and combined interbody and posterior fusion via posterior-only approach. Eur Spine J.

